# Light-harvesting protein Lhcx3 is essential for high light acclimation of *Phaeodactylum tricornutum*

**DOI:** 10.1186/s13568-018-0703-3

**Published:** 2018-10-23

**Authors:** Ting-Bin Hao, Tao Jiang, Hong-Po Dong, Lin-jian Ou, Xiang He, Yu-Feng Yang

**Affiliations:** 1 Key Laboratory of Eutrophication and Red Tide Prevention of Guangdong Higher Education Institutes, College of Life Science, Jinan University, Guangzhou, 510632 China; 2 School of Ocean and Meteorology, Guangdong Ocean University, Zhanjiang, 524088 China; 3 Yellow Sea Fisheries Research Institute, Chinese Academy of Fishery Sciences, Qingdao, 266071 China

**Keywords:** *Lhcx3*, Overexpression and knockdown, Nonphotochemical quenching, Photoprotection

## Abstract

The light-harvesting protein complexes (Lhc) play key roles in the processes of light absorption and protection in diatoms. However, different Lhc protein carries out distinct function in photosynthesis. For now, roles of many Lhc proteins in light acclimation are largely unknown. Here, function of *Lhcx3* in marine diatom *Phaeodactylum tricornutum* was examined by using reverse genetic technologies. The overexpression of *Lhcx3* led to increased diadinoxanthin + diatoxanthin content and elevated non-photochemical fluorescence quenching (NPQ) while knockdown of *Lhcx3* reduced NPQ level. In addition, the expression of *Lhcx3* could be induced by blue light but not by red light. After addition of the photosynthetic inhibitor, upregulation of *Lhcx3* transcript in high light could be inhibited by NH_4_Cl, but not by DCMU (3-(3,4-dichlorophenyl)-l,l-dim ethylurea). In contrast, DCMU addition increased expression of *Lhcx3* in high light. In combination with changes of NPQ after addition of inhibitor, we concluded that the *Lhcx3* played key roles in high light acclimation of diatoms. This finding will provide new clues for genetic improvement of *P. tricornutum* with an aim to cultivate new strains with high growth rate.

**Electronic supplementary material:**

The online version of this article (10.1186/s13568-018-0703-3) contains supplementary material, which is available to authorized users.

## Introduction

Diatoms are eukaryotic unicellular microalgae, and widely distributed in various marine environments (Field et al. 1998). They contribute greatly to the global primary production just like terrestrial tropical rain forests and grasslands, and serve as the base of the marine food webs (Armbrust 2009). Diatoms exhibit optimal photosynthetic activity over an extensive range of environments (Litchman et al. 2009). Their excellent adaptive capacity to changing conditions is regarded as a key reason why diatoms are predominant in contemporary oceans (Field et al. 1998). Levels of nonphotochemical quenching (NPQ) of diatoms under high light are much higher than those of higher plants (Roháček et al. 2014), showing their higher ability to dissipate excess energy (Ruban et al. 2004).

The key mechanism for the modulation of diatom photosynthesis is NPQ (Ruban and Murchie 2012). It occurs in the light-harvesting complex (Lhc) of PSII (Ruban and Murchie 2012). Under high light stress, excess light energy could be dissipated by some Lhc proteins as heat (Opačić et al. 2014). Lhc proteins in diatoms are also called fucoxanthin–chlorophyll proteins (Fcps). The Fcps can be divided into three groups: Lhcf, Lhcr, and Lhcx. Similar to the LhcSR proteins of *Chlamydomonas reinhardtii*, Lhcxs of diatoms have been shown to have the function of photoprotection (Bailleul et al. 2010; Dittami et al. 2010; Gundermann and Büchel 2012; Lepetit et al. 2013; Taddei et al. 2016). Using knockdown *Phaeodactylum tricornutum,* it is found that Lhcx1 plays a pivotal role in managing light responses (Bailleul et al. 2010). More recently, the roles of the other members of the Lhcx family in *P. tricornutum* were studied by using transgenic lines overexpressing the Lhcx proteins, revealing that different overexpressing lines with different Lhcx protein levels had a similar NPQ increase (Taddei et al. 2016). This probably suggests the complexity of NPQ regulation in diatoms. However, detailed roles for Lhcx2, Lhcx3 and Lhcx4 are still obscure.

At present, the genomes of *P. tricornutum* are publicly available (Bowler et al. 2008). *P. tricornutum* has been considered representative model species for studying response mechanism of diatoms to environmental changes, and also is one of the promising oleaginous algal species for biofuel production (Balamurugan et al. 2017; Wang et al. 2015). It is very important for lipid production of *P. tricornutum* to increase its biomass by regulating light-harvesting capacity. The *Lhcx3* gene in *P. tricornutum* was found to be markedly upregulated in high light stress along with the increase of NPQ (Lepetit et al. 2013), suggesting that Lhcx3 took part in high light acclimation and photoprotection. But, to date, no evidence from reverse genetics directly supports the conclusion. Here, we showed that *Lhcx3* played an important role in acclimation response of diatoms to high light by altering expression of *Lhcx3*. This finding will provide new clue for genetic improvement of *P. tricornutum* in efficiency of light energy utilization.

## Materials and methods

### Strain and culture conditions

*Phaeodactylum tricornutum* Bohlin CCMP-2561 was purchased from the Provasoli-Guillard National Center for Marine Algae and Microbiota, USA. *P. tricornutum* was incubated in natural seawater supplemented with f/2 (Guillard and Ryther 1962) without Na_2_SiO_3_∙9H_2_O. Microalgae were cultured in a climate incubator at 20 ± 0.5 °C under 12:12 h photoperiod with irradiance of 30 μmol m^−2^ s^−1^. High light (HL) treatments were implemented by irradiating the cells with 800 μmol m^−2^ s^−1^ for 6 h, then darkness treatments for 1 h. For experiments of light quality, cells were illuminated with monochromatic blue light (BL, 450 nm) or red light (RL, 520 nm). Low light (30 μmol m^−2^ s^−1^, LL), low medium light (100 μmol m^−2^ s^−1^, LML), and medium light (200 μmol m^−2^ s^−1^, ML) were used for BL or RL treatment. For experiments of inhibitor treatment, NH_4_Cl (5 mM, in water) and (3-(3,4-dichlorophenyl)-1,1-dimethylurea) DCMU (10 μM, in ethanol; final concentration of ethanol, 2‰) were added 20 min before starting the HL exposure. Cell density was counted by Brightline hemocytometer under an optical microscope. Cultures were collected at late log phase (day 7) for the following experiments.

### Cloning, vector construction and algal transformation

The *Lhcx3* mRNA sequence of *P. tricornutum* was retrieved from NCBI database (http://www.ncbi.nlm.nih.gov/BLAST/). Phylogenetic analysis was performed by ClustalW2 and the phylogenetic tree was constructed by MEGA7.0 using neighbor-joining method. Total RNA from *P. tricornutum* was extracted using a plant RNA isolation kit (Omega, USA) and transcribed into cDNA using PrimeScriptTM RT reagent Kit with gDNA Eraser (Takara, Japan) according to the manufacturer’s instruction. The full-length cDNA of *Lhcx3* was PCR amplified using the Lhcx3-f/Lhcx3-r primer set. RNA knockdown vectors were constructed according to the method described by De Riso et al. (2009). Briefly, two fragments (short: 247 bp, corresponding to the *PtLhcx3* gene sequence from 268 bp to 514 bp; long: 365 bp, corresponding to the *PtLhcx3* gene sequence from 268 bp to 633 bp) of the full length *PtLhcx3* cDNA were PCR amplified. All primers are provided in Additional file 1: Table S1.

**Fig. 1:**
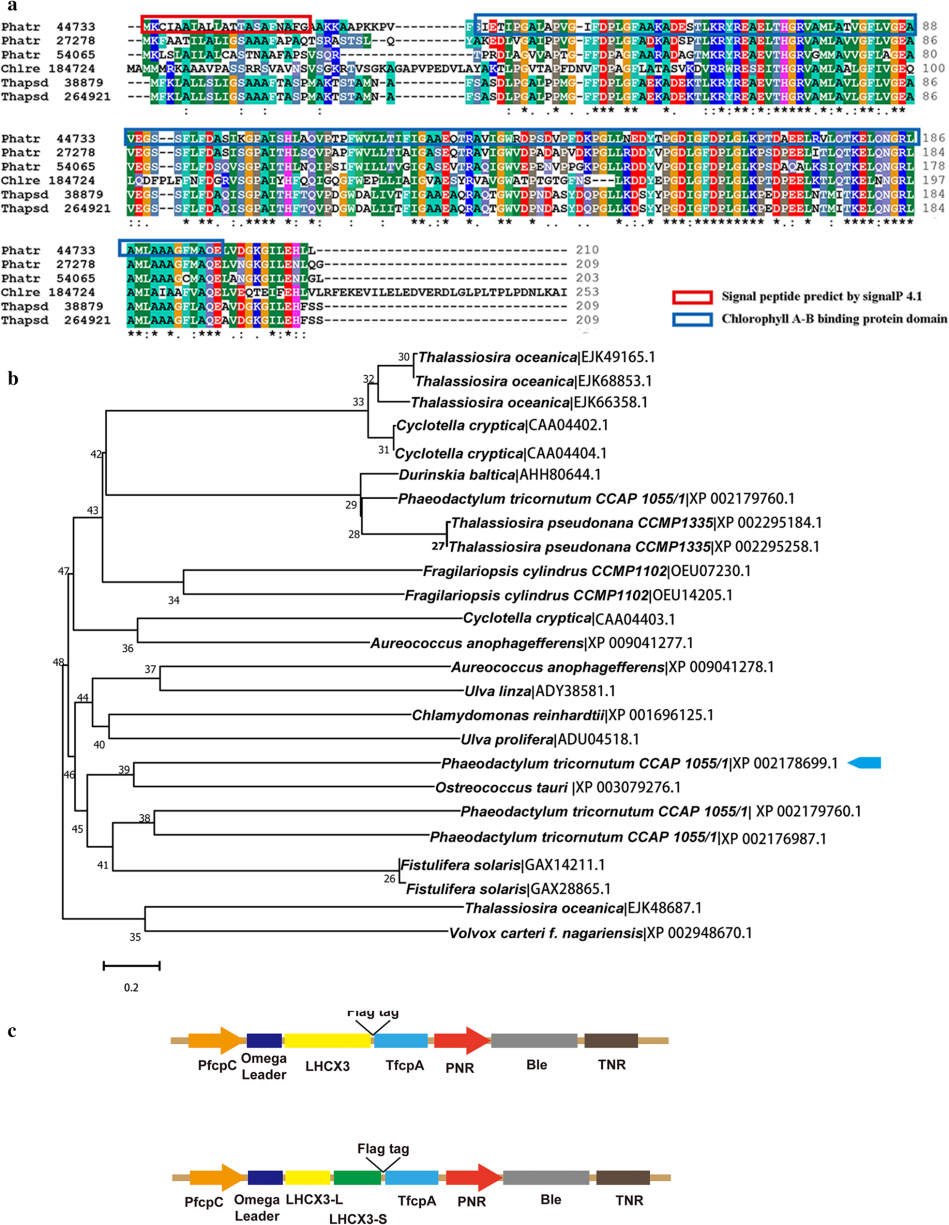
Amino acid sequence analysis of *PtLhcx3* and design of *PtLhcx3* expression construct. **a** The conserved domain of *PtLhcx3* amino acid sequence in *P. tricornutum*. The amino acid sequences of *Lhcx1* (Phatr 27278) and *Lhcx2* (Phatr 54065) from *P. tricornutum*, *Lhcx2* (Thapsd_38879) and *Lhcx LI818* (Thapsd 264921) from *T. pseudonana*, and LI818 (Chlre 184724) from *Chlamydomonas reinhardtii* were shown. **b** Phylogenetic tree showing evolutionary position of *PtLhcx3*. The top 25 hit were used as reference sequences when amino acid sequence of *PtLhcx3* was searched against non-redundant protein database by BLASTP. Phylogenetic tree was constructed using the Neighbor-Joining method in MEGA7. The phylogenetic tree was tested by bootstrapping with 500 replicates and the percentages of bootstrap support were given on the branches. The PtLhcx3 was indicated by the blue arrow. **c** The schematic map showing cassettes of *PtLhcx3* overexpression and knockdown driven by PfcpC promoter. An omega-leader was used for the enhancement of transcription

Vector for *Lhcx3* overexpression was generated by cloning the full-length cDNA sequence of the *Lhcx3* gene into the Phy-21 vector (Siaut et al. 2007). The 633-bp fragment of *Lhcx3* was purified and cloned into the diatom expression vector using the ClonExpress II one step kit (Vazyme, China), which yielded a recombinant expression vector Phy21-*PtLhcx3*. The *Lhcx3* cDNA was inserted between the fucoxanthin chlorophyll a/c binding protein (fcp) fcpC promoter and fcpA terminator of *P. tricornutum*. Furthermore, in order to enhance transcriptional efficiency, an Omega leader sequence was placed before the coding region of *PtLhcx3* (Fig. 1c). The recombinant vector was electroporated into *P. tricornutum* as previously described (Wang et al. 2015). Transformants were selected and maintained in f/2 medium supplemented with 30 μg mL^−1^ bleomycin (Ble).

### Detection of transformants

The *Ble* gene in transformants was detected by PCR using the Ble-f/Ble-r primer set (Additional file 1: Table S1). The relative abundance of *PtLhcx3* transcript was quantified by quantitative real-time PCR (qRT-PCR) using SYBR Green qPCR SuperMix (Invitrogen, USA). Total RNA was isolated using Trizol (Invitrogen, USA), and reversely transcribed into first strand cDNA using Promega Reverse Transcription System (Promega, USA) with random primers according to the manufacturer’s instruction. The qPCR was performed in 8-strip PCR tubes on ABI PRISM^®^ 7500 Sequence Detection System (ABI, USA). The relative gene expression was measured by the 2^−ΔΔCt^ method after normalized to the internal reference gene, β-actin. The qPCR primers are provided in Additional file 1: Table S1.

For Western blot analysis, total protein was extracted using the Total Protein Extraction Kit (Vazyme, China). The protein content was quantitated by the BCA Protein Quantification Kit (Beyotime, China). Protein separation and electrotransfer were performed following standard procedures. Afterwards, the blot was incubated with primary anti-FLAG-tag antibody (1:3000, Abcam, USA) and HRP-conjugated goat anti-rabbit secondary antibody (1:5000, CST, USA), respectively. The membrane was developed using the chemiluminescent system. Endogenous β-actin was used as reference protein.

### Chlorophyll fluorescence measurements

The chlorophyll fluorescence parameters of the transgenic and wild type (WT) cells were measured using PhytoPAM Phytoplankton Analyzer (WALZ, Germany) at room temperature. Effective quantum yield of PSII (*Φ*_*PSII*_) and relative electron transport rate (rETR) were read directly from PhytoPAM. NPQ was calculated using the formula: NPQ = (Fm – Fm′)/Fm′ (Bilger and Björkman 1990), where Fm and Fm′ are the maximum fluorescence emission levels in the dark and light-acclimated cells, respectively.

#### Detection of the reactive oxygen species (ROS) in cells

Intracellular ROS was detected using the dye dihydrorhodamine 123 (DHR123, Molecular Probes, Abcam) following a previous method (Jamers et al. 2009). Briefly, 100 μM DHR123 stock solutions were prepared in DMSO. And then, 10 μM DHR123 was freshly prepared in deionized water from stock solution before staining. Algal cells were incubated at room temperature for 20 min in the dark with DHR123 working solution. These stained cells were observed under a laser-scanning confocal microscope LSM880 (Zeiss, Germany) with an excitation wavelength of 507 nm, an emission wavelength of 529 nm.

### Pigment analysis

For pigment analysis, 50 ml of culture was filtered on GF/F filter and the filter was immediately frozen in liquid nitrogen, and stored at − 80 °C until analysis. Pigment quantitation was performed using an Agilent 1200 HPLC system (Agilent technologies, USA) with a Symmetry C8 column as described previously (Jakob et al. 1999).

### Statistical analysis

All experiments were performed at least in triplicate and the results were described as mean ± SD (standard deviation of the mean). Statistical comparison between transgenic lines and wild type was performed using Student’s *t* test. Difference with *P* < 0.05(*) or *P* < 0.01(**) was considered statistically significant.

## Results

### Sequence analysis of Lhcx3 in P. tricornutum

Amino acid sequences of the *Lhcx3* (Phatr 44733) contained a conserved domain of chlorophyll a/b-binding which was predicted by InterPro (http://www.ebi.ac.uk) and indicated by blue frame in Fig. 1a. The signal peptide predicted by Signal P 4.1 was shown in red frame. Small difference of amino acid could be observed for the conserved domain of chlorophyll a/b-binding by aligning with other *Lhcx* proteins. Phylogenetic analysis showed that the *PtLhcx3* (XP 002178699.1) had high similarity with the protein (XP 003079276.1) of *Ostreococcus tauri* (Fig. 1b). Furthermore, we predicted subcellular localization of the *PtLhcx3* by using LocTree3, showing that the *PtLhcx3* was localized to chloroplast membrane. In order to reveal function of *Lhcx3*, RNAi and overexpression vectors including promoter, 5′ leader, selectable marker were constructed (Fig. 1c).

### Responses of PtLhcx3 to light quality and photosynthetic inhibitors

**Fig. 2:**
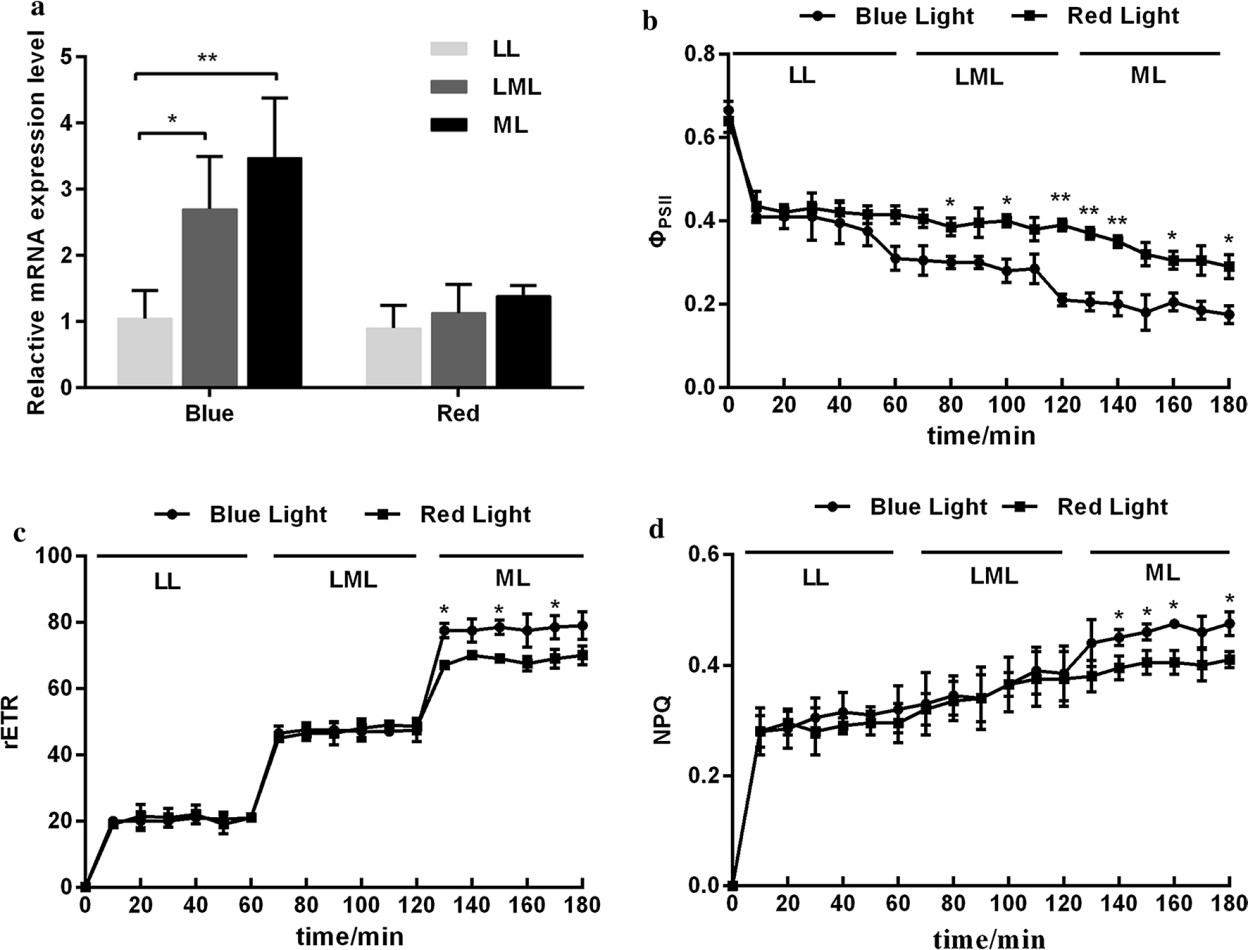
Responses of *Lhcx3* (**a**), *Φ*_PSII_ (**b**), rETR (**c**) and NPQ (**d**) of *P. tricornutum* to blue or red light. LL, LML, and ML represent the light intensities of 30 μmol m^−2^ s^−1^, 100 μmol m^−2^ s^−1^ and 200 μmol m^−2^ s^−1^, respectively. rETR is the relative electron translation rate. Significant difference is indicated at *P *< 0.05 (*) or *P* < 0.01 (**) level. Each value represents mean ± SD (n = 3)

Under blue light (BL), the relative abundance of Lhcx3 transcript was significantly increased as light intensity increased, but did not change under red light (RL) (Fig. 2a). In addition, the *Φ*_PSII_ in BL was lower than that in RL, but no significant difference was observed in rETR and NPQ between BL-treated and RL-treated cells when LL or LML was applied (Fig. 2b–d). However, under ML conditions, BL cultures had an increased capacity of NPQ and a larger rETR compared to RL cultures.

**Fig. 3:**
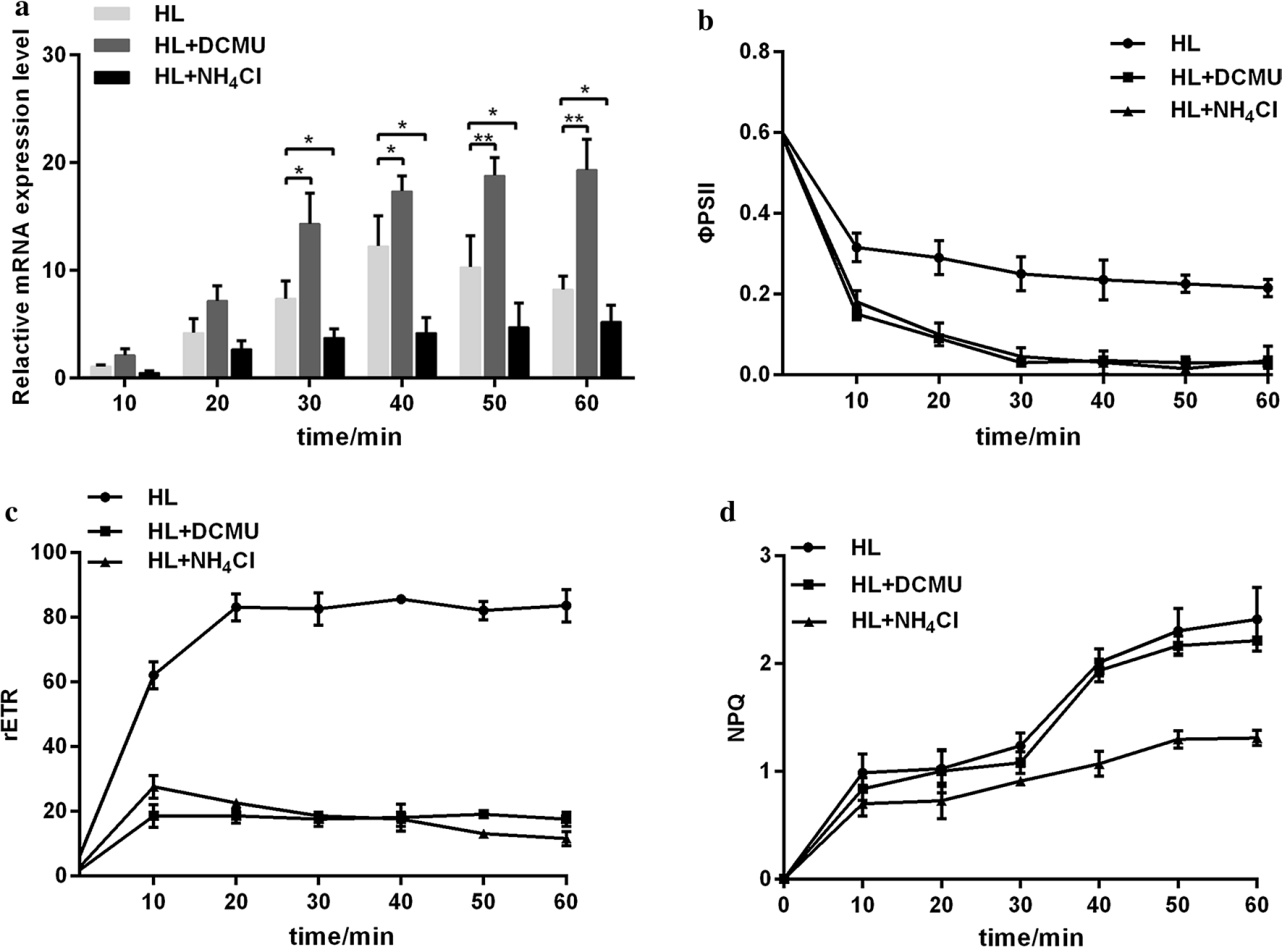
Responses of *Lhcx3* (**a**), *Φ*_PSII_ (**b**), rETR (**c**) and NPQ (**d**) of *P. tricornutum* to inhibitor addition. HL represents the light intensity of 800 μmol m^−2^ s^−1^. Significant difference is indicated at *P* < 0.05 (*) or *P *< 0.01 (**) level. Each value represents mean ± SD (n = 3)

After addition of the photosynthetic inhibitor, upregulation of *Lhcx3* transcript under high light could be inhibited by NH_4_Cl, but not by DCMU (Fig. 3a). In contrast, DCMU addition led to an increased expression of *Lhcx3* gene in high light. In addition, the increase of the rETR in high light (HL) was significantly inhibited by DCMU or NH_4_Cl addition (Fig. 3c). The reduction of the *Φ*_PSII_ in HL was further strengthened by DCMU or NH_4_Cl addition (Fig. 3b). Nevertheless, the NPQ increase in HL was only prevented by NH_4_Cl, but not by DCMU.

### Molecular characterization of transgenic lines

**Fig. 4:**
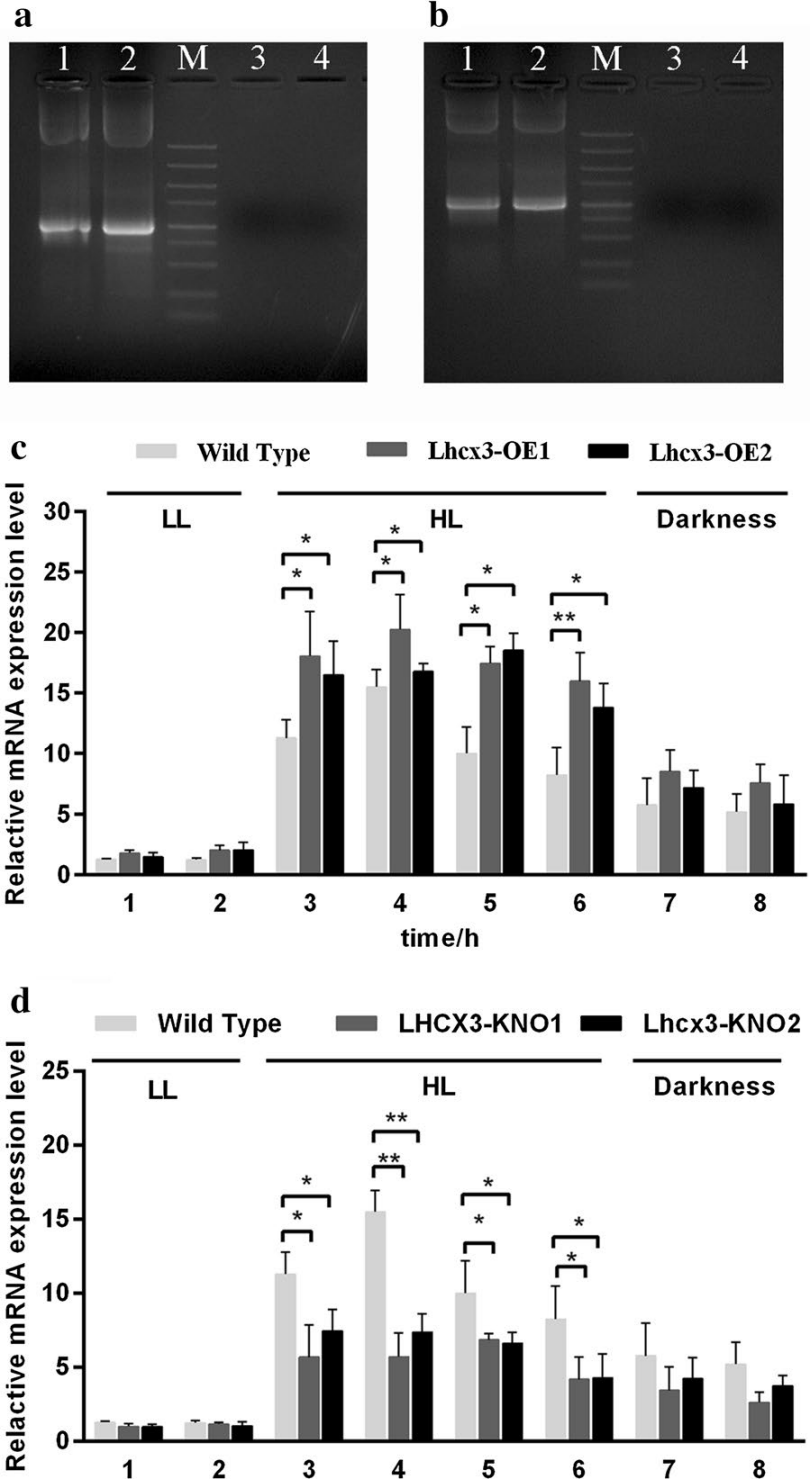
Molecular characteristics of transgenic lines. **a** *Ble* gene in overexpressed lines detected by genomic PCR; Lane M: 5000 bp marker, Lane 1, 2: A 1 kb band corresponding to *Ble* gene; Lane 3: genomic DNA of WT cells; Lane 4: water; **b** *Ble* gene in knocked-down lines detected by genomic PCR; Lane M: 5000 bp marker, Lane 1, 2: A 1 kb band corresponding to *Ble* gene; Lane 3: genomic DNA of WT cells; Lane 4: water; **c** Relative transcript level of *Lhcx3* in overexpressed lines (OE1 and OE2) and wild type. Significant difference between WT and transgenic lines is indicated at *P* < 0.05 (*) or *P* < 0.01 (**) level. Each value represents mean ± SD (n = 3); **d** Relative transcript level of *Lhcx3* in knocked-down lines (KNO1 and KNO2) and wild type. LL = 30 μmol m^−2^ s^−1^, HL = 800 μmol m^−2^ s^−1^

A 1-kb *Ble* gene fragment was PCR amplified from genomic DNA of transgenic cells, whereas no such fragment was detected in WT (Fig. 4a, b). This data showed that the expression cassette of the pHY21 has been successfully integrated into the host genome. qPCR analysis indicated that during HL, a significant increase and decrease were observed in relative abundance of *Lhcx3* transcript in overexpressed (OE) and knocked-down (KNO) lines, respectively, compared to that of WT (Fig. 4c, d).

**Fig. 5:**
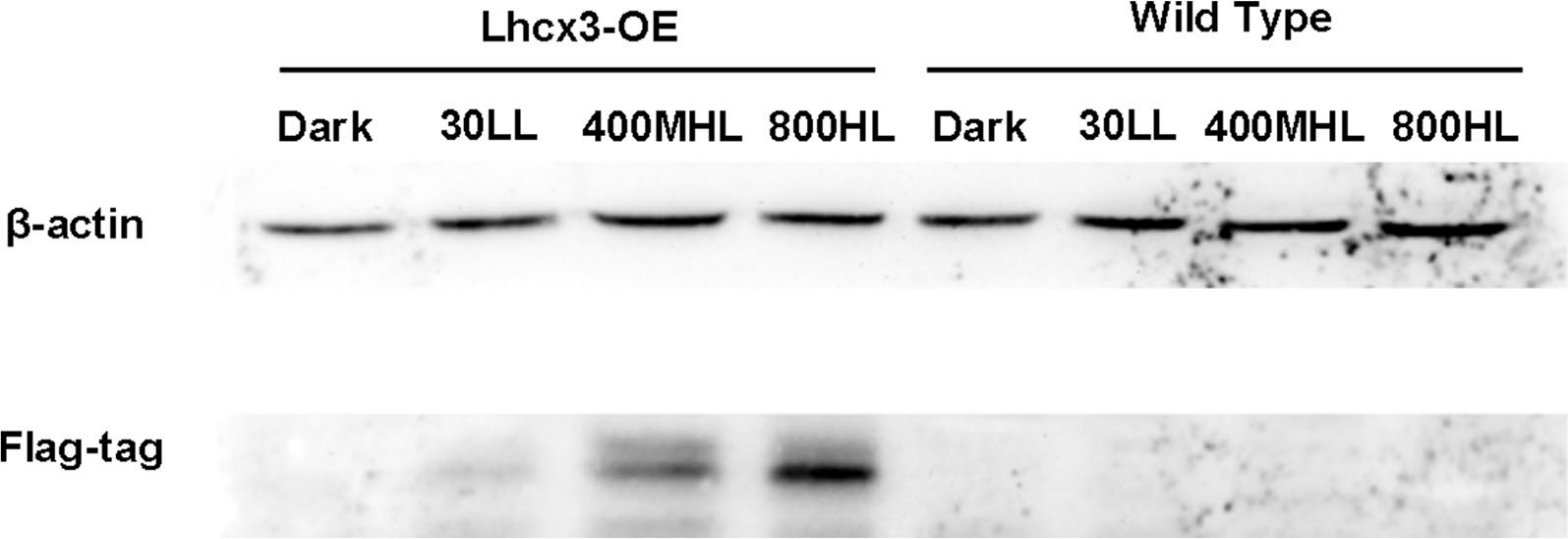
Western blot analysis of FLAG-tag in WT and *Lhcx3*-overexpressed lines (Lhcx3-OE) under different light treatments. β-actin was used as an internal control. LL = 30 μmol m^−2^ s^−1^, MHL = 400 μmol m^−2^ s^−1^, HL = 800 μmol m^−2^ s^−1^

In addition, we detected expression of FLAG-tag ligated to the *PtLhcx3* in overexpression vector in OE lines by using Western blot (Fig. 5). A specific cross-reactive band was observed in OE lines, whereas no band was detected in WT. We also observed that expression of FLAG-tag was increased gradually as light intensity went up from 30 to 800 μmol m^−2^ s^−1^ while no expression occurred in OE lines in the dark.

### Phenotype changes of transgenic lines

**Fig. 6:**
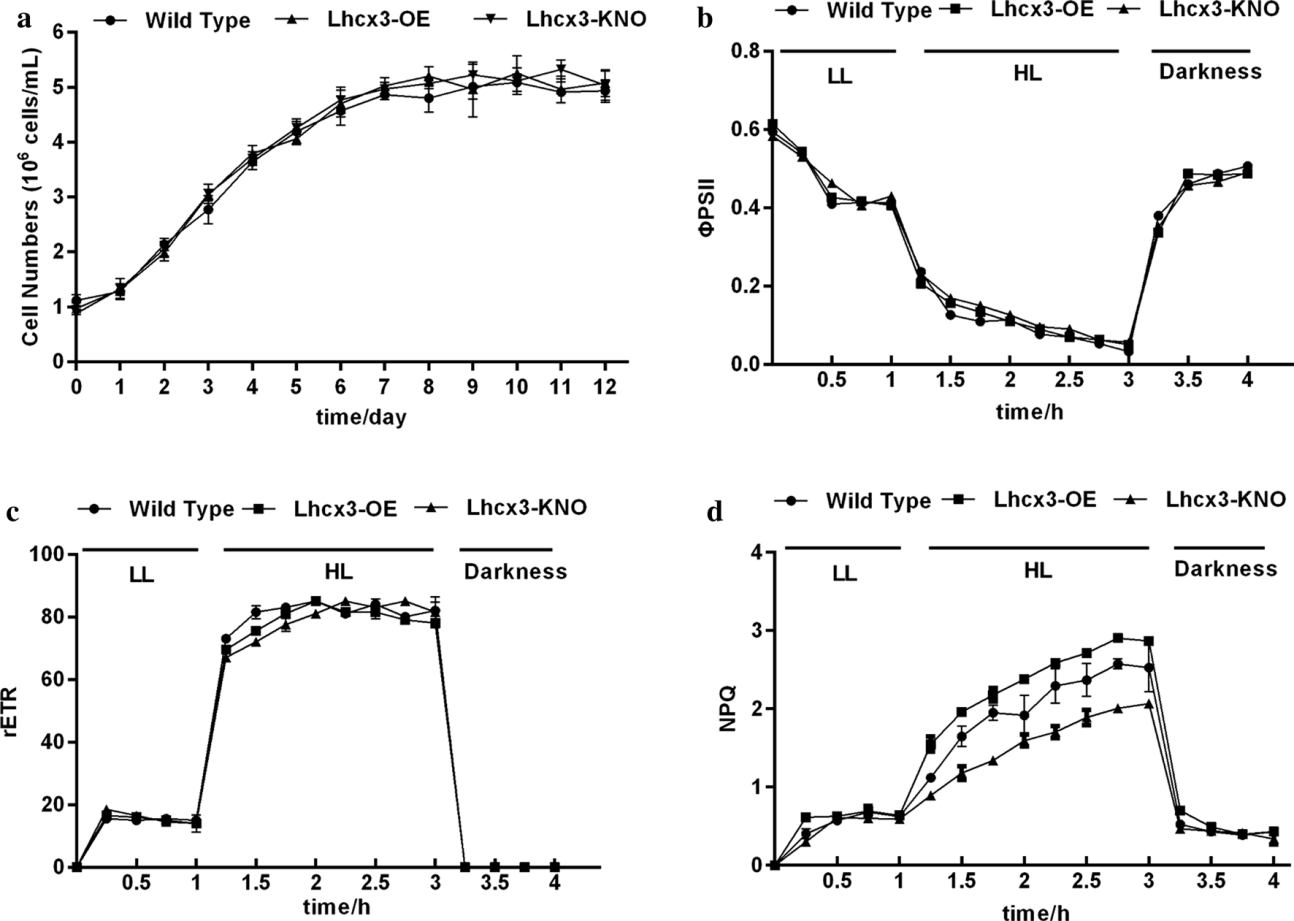
Growth curves (**a**), *Φ*_PSII_ (**b**), rETR (**c**) and NPQ (**d**) of transgenic lines. Significant difference between wild type and transgenic microalgae is indicated at *P* < 0.05 (*) or *P *< 0.01 (**) level. Each value represents mean ± SD (n = 3). LL = 30 μmol m^−2^ s^−1^, HL = 800 μmol m^−2^ s^−1^

**Fig. 7:**
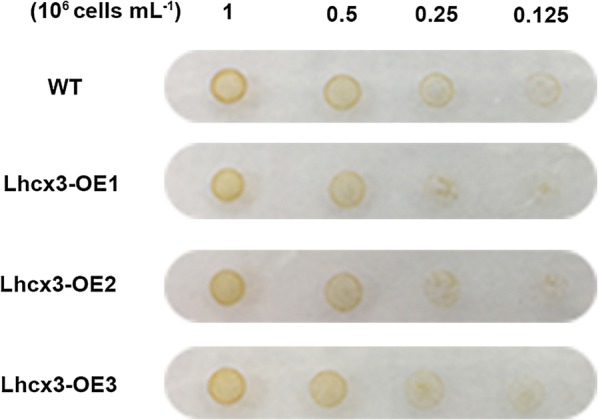
Growth tests on cells spotted on solid f/2 media. A total of 5 μL of cell dilutions (1, 0.5, 0.25, and 0.125·10^6^·mL cells) were spotted and images were taken after 5 days

To analyze the effect of *PtLhcx3* overexpression and knockdown on cellular physiological characteristics of transgenic lines, we determined the growth and chlorophyll fluorescence of the transgenic lines. Compared to WT cells, no significant difference was observed in growth rate, *Φ*_PSII_ and rETR in *PtLhcx3*-overexpressed and knocked down lines in LL, HL or darkness (Figs. 6a–c and 7). However, during HL treatment, overexpression of *PtLhcx3* led to an increased NPQ while knockdown of *PtLhcx3* reduced NPQ (Fig. 6d).

**Fig. 8:**
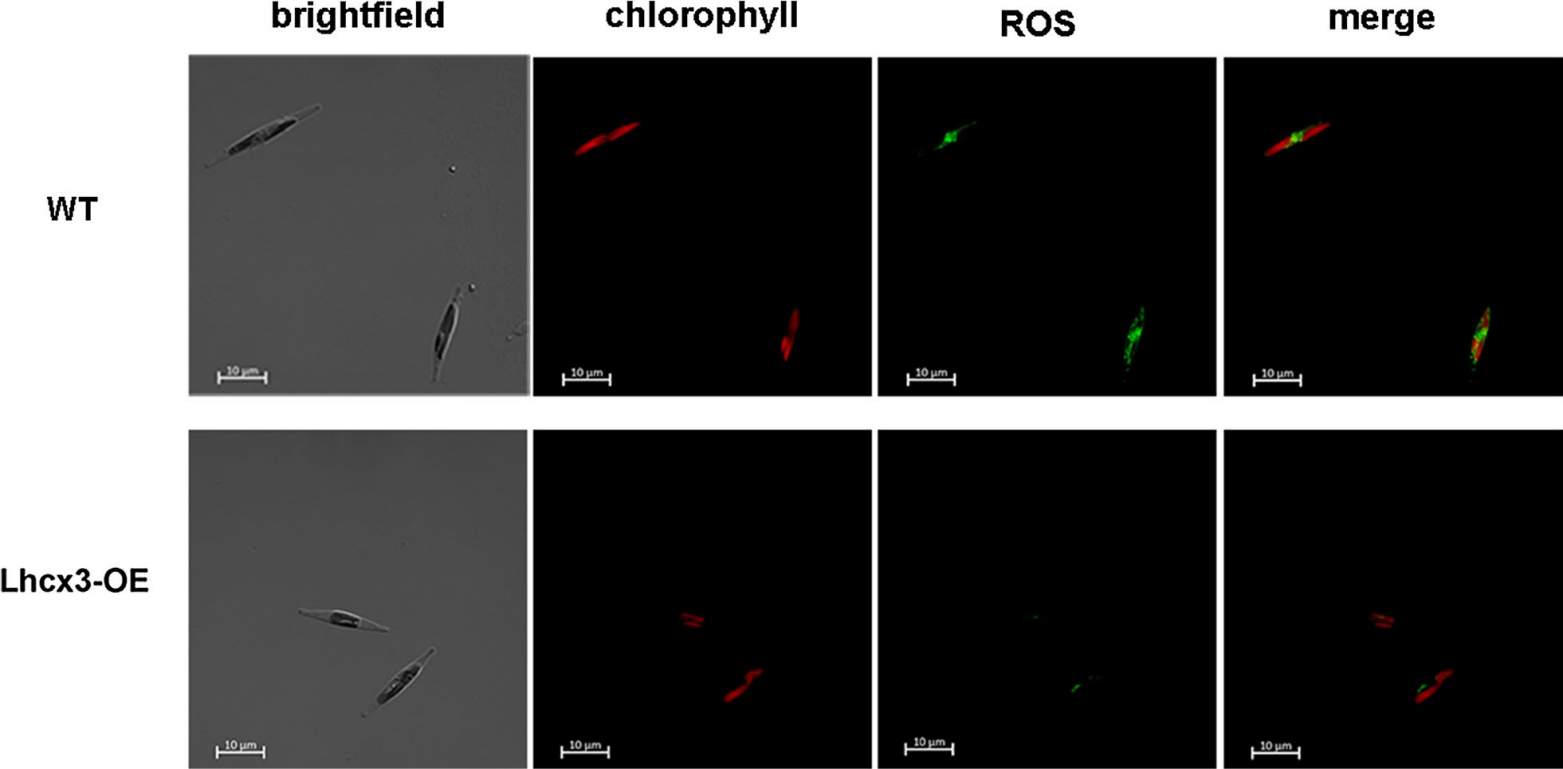
Confocal microscopy images of wild type (WT) cells and *PtLhcx3* overexpression lines for detecting reactive oxygen species (ROS). From left to right, Brightfield; chlorophyll autofluorescence; ROS fluorescence; flurescence overlay. Scale bars represent 10 μm

To evaluate capacity to dissipate excess light energy in OE lines, we detected the level of ROS. ROS is induced by high light stress and its level can be measured using the ROS-sensitive dye, DHR123. ROS can oxidize DHR123 into the fluorescent derivative rhodamine 123. As shown in Fig. 8, obvious fluorescent signals were observed in WT cells, whereas they were hardly detected in OE lines.

The diadinoxanthin (Dd) and diatoxanthin (Dt) content was analyzed in WT and OE lines under LL or HL treatment (Table 1). Whether in LL or HL, both Dd and Dt content in OE lines was significantly higher than those in WT. However, after 1 h of HL exposure, the percentage of Dt was increased significantly in OE lines compared to WT while the percentage of Dd was reduced significantly.

**Table 1:** The content of diadinoxanthin (Dd) and diatoxanthin (Dt) in WT and OE lines after LL-acclimated cells were subjected to 1 h of HL treatment

Strains	Treatment	Dd μg/cell (10^−9^)	Dt μg/cell (10^−9^)	(Dd + Dt)μg/cell (10^−9^)	Dd/(Dd + Dt) (%)	Dt/(Dd + Dt) (%)
Wild type	LL	23.08 ± 1.09	2.00 ± 0.11	25.08 ± 1.09	92.02 ± 0.56	7.98 ± 0.56
	HL	20.33 ± 1.49	3.50 ± 0.16	23.82 ± 1.57	89.96 ± 1.75	11.04 ± 1.75
Lhcx3- OE	LL	32.79 ± 2.04**	3.91 ± 0.73**	36.70 ± 1.32**	89.29 ± 2.32	10.71 ± 2.32
	HL	33.60 ± 2.75**	5.49 ± 0.23**	39.09 ± 2.55**	84.22 ± 1.23**	16.78 ± 1.12**

Data represent the means of three independent samples ± standard deviations. Asterisks represent significant differences (*P *< 0.05). HL- or LL-treated OE lines were compared with HL- or LL treated WT, respectively. LL = 30 μmol m^−2^ s^−1^, HL = 800 μmol m^−2^ s^−1^

## Discussion

Diatoms have excellent capability to deal with changing light intensity in complicated marine environment. However, it is unclear how diatoms acclimate to drastic fluctuations in light intensity. For now, the Lhc proteins have been shown to play key roles in the processes of light absorption and protection in photosynthetic organisms (Govindjee 2002; Mozzo et al. 2008). Different Lhc proteins possess distinct functions. Thus, it is necessary to uncover roles of Lhc proteins in photosynthesis for providing new insights into processes of light energy utilization in diatoms.

Western-blot analysis of FLAG-tag indicated that *Lhcx3* has been successfully expressed in OE lines. We also observed that LL, MHL or HL could induce expression of FlAG-tag, but band intensities were different, showing that the fcpC promoter was controlled by light. Considering that comparisons of physiological parameters between transgenic lines and WT were performed at the same light intensities, the difference of *Lhcx3* expression under different light conditions did not affect their comparison. In transgenic lines with changed expression of *PtLhcx3* under different light conditions, no significant changes were observed in the growth rate, *Φ*_PSII_ and rETR compared to the WT, suggesting that *PtLhcx3* might have nothing to do with cell growth and photosynthetic activity. In contrast, NPQ was significantly elevated or decreased in transgenic lines under HL, suggesting that *PtLhcx3* may participate in photoprotection to prevent diatom cells from the damage of excess light. It has been reported that *PtLhcx1* in the Lhcx family played a key role in the dissipation of excess light energy (Bailleul et al. 2010). But expression pattern of *PtLhcx1* was different from that of *PtLhcx3*, it could not be induced by HL and was kept a high transcriptional level during the whole course of growth regardless of light conditions (Nymark et al. 2009). On the contrary, *PtLhcx3* can be induced by HL. In photosynthesis, photosystems II (PSII) electrolyze H_2_O to produce proton gradient under HL and the LHC become aggregated due to the protonation of LHC and the de-epoxidation of diadinoxanthin to diatoxanthin (Goss et al. 2006). The aggregated LHC can dissipate excess light energy as heat to protect the photosynthetic apparatus from damage induced by HL (Walters et al. 2010). The induction of *PtLhcx3* under HL might play a key role in triggering assembly of LHC.

The *PtLhcx3* could be induced by BL, not by RL. Meanwhile, an increased NPQ was observed in BL cultures under ML conditions compared to RL cultures. A previous study found that *P. tricornutum* cultures grown under BL had an increased NPQ and a larger pool of xanthophyll cycle pigments compared to cultures grown under white light and RL (Costa et al. 2013), suggesting that BL induced an enhanced photoprotective potential. The upregulation of *PtLhcx3* under BL conditions implicated that *PtLhcx3* might be involved in photoprotection. Moreover, blue light receptor proteins such as CPF1 (Coesel et al. 2009) and CRYP (Juhas et al. 2014) have been found in *P. tricornutum*, and they may transmit light signal to regulate the expression of *PtLhcx3*.

After the addition of DCMU, the level of *PtLhcx3* transcript in HL was significantly increased. DCMU is an inhibitor of electron transport chain of PSII, which can imitate a low light environment (Ridley and Horton 1984). When electron transfer was blocked by DCMU, electron accumulation will produce ROS in thylakoid (Lepetit et al. 2013). It is presumed that ROS induced the increase of *PtLhcx3* transcript in HL. Notably, the addition of DCMU did not inhibit the increase of NPQ in HL, suggesting that *PtLhcx3* might contribute to NPQ elevation. The reduced level of ROS in *PtLhcx3* overexpression lines also supported the notion. The NH_4_Cl addition not only inhibited the level of *PtLhcx3* transcript, but also reduced the increase of NPQ in HL. NH_4_Cl can remove the ΔpH of both sides of thylakoid membrane directly (Lovyagina and Semin 2016) while the induction of NPQ depends on the ΔpH (Eisenstadt et al. 2010). For now, it is still unclear how ΔpH induces NPQ. However, if sufficient *PtLhcx3* protein is required for the development of NPQ, it is likely that changes of pH value in lumen of thylakoid affected the expression of *PtLhcx3*, which conversely influenced the formation of NPQ.

Compared to WT, both Dd and Dt content was raised significantly in OE lines regardless of light intensities. Meanwhile, after 1 h of HL exposure, Dt/(Dd + Dt) in OE lines was significantly higher than that in WT. These results suggested that increased expression of *Lhcx3* in cells elicits de novo synthesis of the Dd + Dt pool. In *T. pseudonana*, it has been suggested that *Lhcx6* was combined with Dt and participated directly in response of diatoms to HL (Zhu and Green 2010). Here, we hypothesized that the Dt in *P. tricornutum* may be combined with the *Lhcx3*. In this case, increased *Lhcx3* required inevitably more Dt, which may provoke synthesis of more Dd for maintaining conversion between Dt and Dd. Given that Dt is vital for maintaining high level of NPQ during HL treatment (Eisenstadt et al. 2010), it is reasonable that an elevated NPQ occurred in Lhcx3-overexpressed lines. The results indicate there is a tight linkage among *Lhcx3*, Dt and NPQ.

## Additional file

**Additional file 1: Table S1.** List of primers used in this study.
