# Supplementary material for: Light-harvesting protein Lhcx3 is essential for high light acclimation of Phaeodactylum tricornutum
Source: AMB Express. 2018 Oct 23;8:174. doi: 10.1186/s13568-018-0703-3 (PMC6199207; doi:10.1186/s13568-018-0703-3)
Supplement: Supplementary file 1 — Additional file 1: Table S1. List of primers used in this study. [file 13568_2018_703_MOESM1_ESM.doc]

**AMB Express**

Light-harvesting protein Lhcx3 is essential for high light acclimation of *Phaeodactylum tricornutum*

Ting-Bin Hao^1^, Tao Jiang^3^, Hong-Po Dong*^2^, Lin-jian Ou*^1^, Xiang He^1^, Yu-Feng Yang^1^

^1^Key Laboratory of Eutrophication and Red Tide Prevention of Guangdong Higher Education Institutes, College of Life Science, Jinan University, Guangzhou 510632, China;

^2^School of Ocean and Meteorology, Guangdong Ocean University, Zhanjiang 524088, China;

^3^Yellow Sea Fisheries Research Institute, Chinese Academy of Fishery Sciences, Qingdao 266071, China

* Corresponding author. donghongpo2001@hotmail.com (H.P. Dong); torangeou@jnu.edu.cn (L.J. Ou) Tel: 0860759-2396055

Table S1 List of primers used in this study

| Gene name | Primers |
| --- | --- |
| PtLhcx3- full-length | F: ATGAAGTGCATCGCCGCTAT |
|  | R: AAGTGTAGTAAGGATTCCTTTATAGTCGTG |
| PtLhcx3-long | F: GAAGGATCTTCCTTCCTCTTTGATGCT |
|  | R: CACGTTTTTCGTGGCGACCA |
| PtLhcx3-short | F: CTTCCGCATCCGTTGGCTT |
|  | R: GAAGGATCTTCCTTCCTCTTTGATGC |
| Bleomycin (Ble) | F: ACCATGGCCAAGCTCACTTC |
|  | R: TTAGTCCTGCTCCTCAGCCAC |
| PtLhcx3-qPCR | F: GAGTCCACCCTGAAGCGATA |
|  | R: GGTCAAGAGAACCCAGAACG |
| Actin | F: TGTGCGTGACATCAAGGA |
|  | R: GCCAATCACGATCACGTTT |
